# Transcriptome Analysis of *Psacothea hilaris*: De Novo Assembly and Antimicrobial Peptide Prediction

**DOI:** 10.3390/insects11100676

**Published:** 2020-10-05

**Authors:** Joon Ha Lee, Hoyong Chung, Yong Pyo Shin, In-Woo Kim, Sathishkumar Natarajan, Karpagam Veerappan, Minchul Seo, Junhyung Park, Jae Sam Hwang

**Affiliations:** 1 Department of Agricultural Biology, National Institute of Agricultural Sciences, 166, Nongsaengmyeong-ro, Iseo-myeon, Wanju-gun, Jeollabuk-do 55365, Korea; coover@korea.kr (J.H.L.); shinyp2020@korea.kr (Y.P.S.); kiw0601@korea.kr (I.-W.K.); nansmc@korea.kr (M.S.); 2 3BIGS CO. LTD., 156, Gwanggyo-ro, Yeongtong-gu, Suwon-si, Gyeonggi-do 16506, Korea; hychung@3bigs.com (H.C.); sathish@3bigs.com (S.N.); karpagam@3bigs.com (K.V.)

**Keywords:** *Psacothea hilaris*, knottin, psacotheasin, antimicrobial peptide, transcriptome, hemolysis

## Abstract

**Simple Summary:**

Multidrug resistance for classical antibiotics is the major problem faced in clinical infections. Insects have antimicrobial peptides (AMPs) which are a part of the immune repertoire to protect it from microbial pathogens. AMPs are short chain protein molecules that function as antibacterial, antifungal, antiparasitic and antiviral factors. Insect AMPs are present in their hemolymph or produced in response to entry of pathogens and kill them to protect the host. The objective of this study is to identify novel AMPs from *Psacothea hilaris*, the yellow spotted long horned beetle. The beetle was immunized with bacteria and fungi and RNA was isolated. The RNA was processed and screened by in silico strategies to identify novel AMPs. We obtained one potential candidate which was tested to be effective against harmful bacteria and fungi. This will be useful in treating multidrug resistant microbes alone or in combination with antibiotics with future validations.

**Abstract:**

Antimicrobial peptides (AMPs) are the frontline innate defense system evolutionarily preserved in insects to combat invading pathogens. These AMPs could serve as an alternative to classical antibiotics to overcome the burden of treating multidrug resistant bacteria. Psacotheasin, a knottin type AMP was isolated from *Psacothea hilaris* and shown to exhibit antimicrobial activity, especially against fungi through apoptosis mediated cell death. In this study, we aimed to identify novel probable AMPs from *Psacothea hilaris*, the yellow spotted longicorn beetle. The beetle was immunized with the two bacterial strains (*E. coli and S. aureus*), and the yeast strain *C. albicans*. After immunization, total RNA was isolated and sequenced in Illumina platform. Then, beetle transcriptome was de novo assembled and searched for putative AMPs with the known physiochemical features of the AMPs. A selection of AMP candidates were synthesized and tested for antimicrobial activity. Four peptides showed stronger activity against *E. coli* than the control AMP, melittin while one peptide showed similar activity against *S. aureus*. Moreover, four peptides and two peptides showed antifungal activity stronger than and similar to melittin, respectively. Collectively one peptide showed both antibacterial and antifungal activity superior to melittin; thus, it provides a potent antimicrobial peptide. All the peptides showed no hemolysis in all the tested concentrations. These results suggest that in silico mining of insects’ transcriptome could be a promising tool to obtain and optimize novel AMPs for human needs.

## 1. Introduction

Insects are the largest producer of antimicrobial peptides (AMPs) with distinct characteristics. The habitat of the insects which includes confronting microbes necessarily potentiates it to produce a wide range of AMPs for its survival. Thus, AMPs especially in insects are foreground defense machinery against attacking microbes entering the host. Most of them are cationic, hydrophobic, and amphipathic peptides with < 50 amino acids [1]. These unique features of the AMPs target the microbial cell membrane and increases the permeability, leading to microbial cell death. In the recent years, AMPs gained attention as new drugs or alternate candidates over classical antibiotics to overcome the acquired multidrug resistance of microbes in clinical settings, because the targets of the AMPs are predominantly microbial membrane which allows reduced resistance compared to conventional antibiotics targeting intracellular components [2,3]. Several AMPs have been purified, identified, and studied from the largest class insect of arthropods in the past decades [4,5,6,7].

Knottin type peptides have a cysteine knot formed by the unique disulfide bonds between Cys1-Cys4, Cys2-Cys5, Cys3-Cys6 residues. Apart from antimicrobial effect, knottin displays a wide range of bio functional activities which include protease and enzyme inhibitors, cytotoxic, and anti-HIV agents [8,9,10]. A knottin type novel AMP “psacotheasin” was isolated from *Psacothea hilaris* (*P. hilaris*), the yellow spotted long horned beetle of Asiatic origin [11]. Psacotheasin is reported to be effective in inhibiting the growth of pathogenic Gram-positive, Gram-negative bacterial and fungal strains without hemolytic effect [11]. The antifungal activity on human fungal pathogen *C. albicans* was by pore formation in the fungal plasma membrane and disturbing its permeability. Furthermore, evidences of mitochondrial mediated apoptosis events may likely contribute to the antifungal activity [12,13].

The promising pharmaceutical effect of psacotheasin accelerated our interest to further identify putative AMPs from *P. hilaris*. We utilized next generation sequencing technology, a modern age tool, to divulge putative AMPs [14,15,16]. In silico screening for AMPs was done on transcriptome of the yellow spotted long horned beetle immunized with bacterial and yeast strains. After immunization, total RNA was isolated and sequenced using the Illumina platform. Transcriptome was assembled de novo followed by the AMP prediction pipeline. Furthermore, selected peptides were synthesized and tested for antibacterial activity and toxicity. We identified a candidate AMP with antimicrobial activity which also showed no hemolysis on mouse red blood cells. This AMP could serve as a candidate therapeutic agent which on further validation could prove it a promising clinical applicant.

## 2. Materials and Methods

### 2.1. Microbial Growth Conditions

The bacterial strains *Escherichia coli* (KACC 13821, ATCC 11775) and *Staphylococcus aureus* (KACC 10768, ATCC 25923), and the yeast strain *Candida albicans* (KCTC 7121, ATCC 14053) were purchased from the Korean Agricultural Culture Collection (KACC) and Korean Collection for Type Cultures (KCTC). Both bacteria and yeast were grown overnight in tryptic soy broth (TSB; Difco, Thermo Fisher Scientific, Waltham, MA, USA) at 37 °C, 200 rpm to the stationary phase. Bacteria were re-inoculated in fresh TSB medium and cultivated for 3 h to the log phase. The strains were stored with 15% glycerol at −70 °C.

### 2.2. Immunization of Insects

*P. hilaris* were reared at room temperature with 60% ± 5% relative humidity and a 16/8 light/dark photoperiod cycle. For immunization, each larva of the yellow-spotted longicorn beetle was injected with the mixture of *E. coli*, *S. aureus*, *C. albicans* (1 × 10^6^ colony-forming units) suspended in 100 μL sodium phosphate buffer (10 mM; pH 7.4). Non-immunized control larva was injected with 100 μL sodium phosphate buffer (10 mM; pH 7.4) alone. After maintaining both immunized and non-immunized larvae at 25 °C ± 1 °C for 18 h, total RNA was isolated.

### 2.3. Transcriptome Sequencing, De Novo Assembly and Functional Annotation

Total RNA quality and quantity of each sample were analyzed by a Bioanalyzer 2100 system using an RNA 6000 Nano kit (Agilent technologies, Inc., Santa Clara, CA, USA). Approximately 2 μg of total RNA ((RNA Integrity Number (RIN) > 7.0) of each sample was used to construct transcriptome cDNA libraries using a Truseq Stranded mRNA Prep Kit (Illumina Technologies, San Diego, CA, USA) according to the manufacturer’s instructions. Furthermore, cDNA libraries were sequenced using the Illumina HiSeq 2500 platform to generate 100 bp paired end reads. Initial raw data quality was checked by FastQC (https://www.bioinformatics.babraham.ac.uk/projects/fastqc/). Then, Trimmomatic [17] was used to remove low quality reads, adaptor contents, contaminated reads from raw reads to obtain clean reads. Further, the Trinity program [18] was used for de novo assembly of high-quality reads with default parameters. The redundancy transcripts were identified through Cluster Database at High Identity with Tolerance CD-HIT-EST [19] with an identify threshold of 95% sequence similarity. In addition, a TransDecoder [18] was used to identify the longest open reading frame (ORF) per transcripts and protein fragment for peptide prediction. Furthermore, functional annotations of filtered unigenes were identified through a homology search against Swiss-Prot databases by BLASTX with E-value cut-off of 10^−5^. The assembled unigenes were subjected to gene ontology (GO) enrichment analysis by GOseq Bioconductor package [20] based on the Wallenius non-central hypergeometric distribution with a corrected *p*-value less than 0.05.

### 2.4. Gene Expression Analysis

To identify gene expression and differential gene expression (DEG) analysis, cleaned raw reads were mapped to assembled unigenes with Bowtie [21] using default parameters. The gene transcript abundance was calculated as transcripts per kilobase million (TPM) [22] using the RNA-Seq by the Expectation-Maximization (RSEM) package [23]. The generated read counts of all genes were given as input for identification of differential expressed genes (DEG) using EdgeR [24]. A *p*-value less than 0.05 (*p* < 0.05) and a log2 Fold Change (log2FC) ≥ 1 between control and immunized samples were selected for downstream analysis.

### 2.5. AMP Screening and Prediction

The deduced amino acid sequences were subjected to AMP prediction analysis using a modified in silico strategy. Peptide characteristics of molecular propensity (based on physicochemical properties) and aggregation propensity (in vitro and in vivo) were determined, and AMP prediction was established using a predefined in silico strategy with parameters defined previously [14]. Finally, the AMPs were mapped with the Collection of Anti-Microbial Peptides (CAMP), A Database of Anti-Microbial peptides (ADAM), Antimicrobial Peptide Database (APD) databases [25,26,27] and classified as novel and known AMPs. The BLAST results were filtered with a similarity score ≥ 80. Sequences with observed similarity at the given cutoff values were considered as known AMPs, and others were considered as novel AMPs. Further, putative AMPs were validated using CAMP and ADAM classifiers. Finally, the novel and the known AMPs were manually validated for continuous stretches of amino acids to account for the low-complexity regions and assembly artifacts.

### 2.6. Peptide Synthesis

The final filtered peptides were synthesized using solid-phase peptide synthesis methods at AnyGen Co. Ltd. (Gwangju, Korea). Then, each peptide was purified to > 95% by high-performance liquid chromatography, and the purity was confirmed by mass spectrometry analysis. The peptides were dissolved in acidified distilled water (0.01% acetic acid) and stored at −20 °C until being used in subsequent experiments.

### 2.7. Antimicrobial Activity Assay

The radial diffusion assay was performed to test the antimicrobial activities of peptides, as described previously with slight modifications [28]. In brief, bacteria and yeast strains were grown to the mid-logarithmic phase in TSB at 37 °C and then washed twice with 10 mM sodium phosphate buffer (pH 7.4). A total of 4 × 10^6^ colony forming unit (CFU) was added to 10 mL of an underlay agarose gel (9 mM sodium phosphate, 1 mM sodium citrate, pH 7.4, 1% (w/v) Type I (low electroendosmosis) agarose (Sigma, St. Louis, MO, USA), and 3% TSB (Difco, USA)). The underlay gel was poured into a 100-mm INTEGRIDTM Petri dish. After agarose solidification, 3-mm-diameter wells were punched and 5 μL of each peptide solution was added to each well. Buffer alone was used as a negative control. Plates were incubated at 37 °C for 3 h to allow for diffusion of the peptides. The underlay gel was then covered with 10 mL of nutrient-rich agar overlay (6% TSB and 1% agarose). The antimicrobial activity of a peptide was measured as the diameter of the cleared zone around each well after 12 h of incubation at 37 °C. This experiment was repeated at least 3 times.

### 2.8. Hemolytic Assay

This experiment was approved by the Institutional Animal Care and Use Committee (IACUC) of the National Institute of Agricultural Sciences (approval number: NAS-202014). The hemolytic activity of the peptides was determined by monitoring the release of hemoglobin from mouse erythrocytes at 540 nm. For the hemolytic assay, 20 μL of each peptide solution at a predetermined concentration was added to 180 μL of a 2.5% (v/v) suspension of mouse erythrocytes in phosphate-buffered saline (PBS). Melittin (Sigma), a hemolytic and α-helical peptide isolated from bee venom, were used as the positive control. This mixture was incubated for 30 min at 37 °C, and 600 μL of PBS was then added to each tube. After 3 min of centrifugation at 10,000× *g*, the supernatant was removed, and the absorbance was measured at 540 nm. Evaluations were made from the results of at least three independent experiments, each carried out in triplicate.

### 2.9. Data Availability

All the raw data utilized in this study have been deposited in the National Centre for Biotechnology (NCBI) Sequence Read Archive (SRA) under the accession number SRA1096680.

## 3. Result and Discussion

### 3.1. Transcriptome Sequencing and Assembly

RNA was isolated and sequenced from totally six samples (Three biological replicates for each group, (Non-immunized vs. Immunized)). Overall workflow includes RNA sequencing, de novo assembly and AMP prediction of *P. hilaris* transcriptome. The total number of raw reads obtained in each group is summarized in Table 1. A total of 132,438,330 clean reads were obtained after adapter trimming and low-quality sequences were removed. Secondly, the obtained clean reads were processed with the de novo transcriptome assembly software package “Trinity”. The de novo alignment of the individual sample ranged between 81.16% and 86.32% (Table 1). The assembly resulted in 35,715 unigenes with the average length of 2052 bp and the unigenes > 500 bp was considered for further processing.

### 3.2. Functional Annotations, Species Distribution and Differential Gene Expression Analysis (DEG)

**Figure 1:**
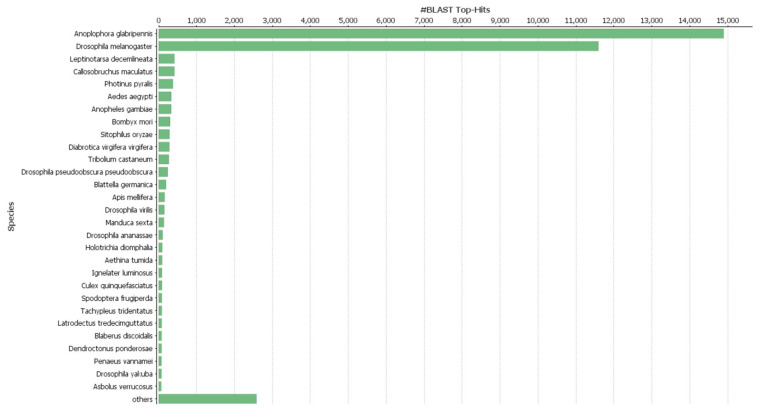
Blast Top hit species distribution match of *Psacothea hilaris* in Arthropoda phylum.

**Figure 2:**
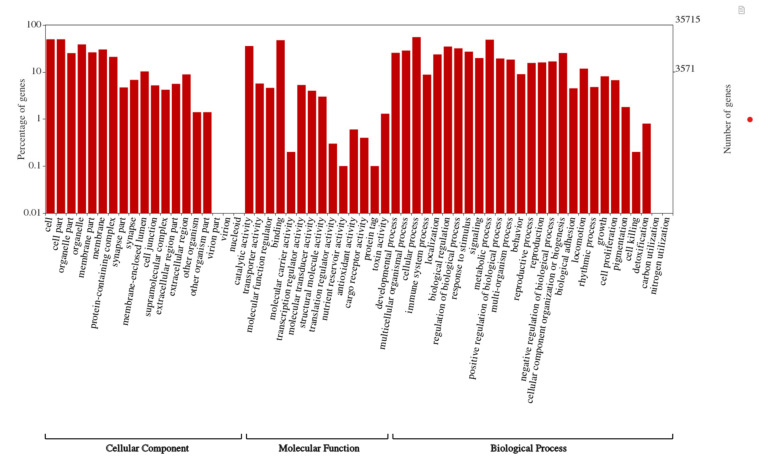
Gene ontology (GO) classification of the *Psacothea hilaris* transcriptome. The GO was summarized into three main categories: Biological process, Cellular component, Molecular function. X axis indicates number of unigenes, and y axis indicates the sub-categories of GO terms.

After the assembly, the unigenes were searched against the Swiss-Prot, and GO databases and annotated. A total of 33,932 (95%) unigenes were annotated by the blastx search against the Swiss-Prot database. The species distribution of the top-hit BLAST against the Swiss-Prot database revealed two closely matched species *viz.*, *A. glabripennis* and *D. melanogaster* (Figure 1). The gene function prediction by GO analysis resulted in 26,492 annotated genes distributed in three major categories, biological process, cellular component, and molecular function (Figure 2). All three categories biological process (22,812), cellular component (21,648), and molecular function (22,970) represented similar number of functional groups. Furthermore, DEG was analyzed to find differences in the expression pattern between immunized and non-immunized *P. hilaris*. The immunization mixture of Gram positive, Gram negative and fungus might potentiate defense pathway activation. A cutoff of log2FC ≥ 2 and a *p*-value < 0.05 was set and a total of 2381 transcripts were obtained to be differentially expressed. Among these, 1206 transcripts were upregulated while 1175 transcripts were downregulated in the immunized group when compared with the control.

### 3.3. AMP Prediction

We aimed to identify putative AMPs from the transcriptome of immunized as well as non-immunized *P. hilaris* utilizing in silico strategies. Previous analysis of AMPs through computation methods has an added scientific insight in understanding AMPs [14,15,16]. Insects are diversified organisms with strong environmental adaptation and a well-built defense system against invading harmful bacteria, fungus, parasites, and viruses [7]. AMPs are multi- targeted which can be competently used to combat the increasing global burden of multidrug resistant bacteria. Psacotheasin, a knottin type AMP isolated from *P. hilaris* was proved to possess antibacterial and antifungal activity [11,12]. The immunized beetle might produce a defensive AMPs which we thrived to study and utilize for human needs. Herein, we used several physiochemical filters obtained from the known AMPs to screen for the possible AMPs. The molecular charge indispensable predictor of physiochemical property is very important so that the AMPs are usually cationic and amphipathic. The aggregation propensity is also necessary so that it does not affect the function of other native proteins. The critical cutoff value and the number of novel AMPs passing through the filters are detailed in Table 2. After the final filters we obtained 2290 AMPs in total, which were again filtered with our inhouse filtering tool. The obtained AMPs were then selected for synthesis and subjected to experimental validations.

### 3.4. Experimental Validation of Putative and Novel AMPs

**Figure 3:**
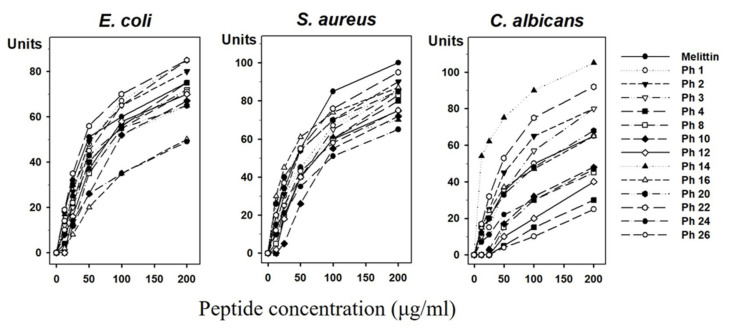
Antimicrobial activity assay. Antimicrobial activities of 13 selected peptides against *E. coli*, *S. aureus*, and *C. albicans* determined by a radial diffusion assay. Peptide concentration (*x*-axis) was plotted against the diameter of the microbial growth inhibition zone (*y*-axis) after incubation for 12 h and is expressed in units (1 mm = 10 units). Melittin was used as a positive control. Mean values were obtained from tests repeated three times.

Experimental validation is required to examine the accuracy of any putative and novel AMPs identified. The 248 peptides were sorted according to the AMPs prediction pipeline, and 193 novel AMPs with potentially high activity were ultimately selected (patent application number: 10-2019-0019906). Among them, 26 peptides were synthesized according to results of the Network Protein Sequence Analysis (NPS^@^) web server (https://npsa-prabi.ibcp.fr/cgi-bin/npsa_automat.pl?page=npsa_gor4.html). We chose the α-helix regions based on secondary structure due to efficiency and cost of peptide synthesis for development of antimicrobial agents. After screening the synthesized peptides, we finally selected 13 AMPs (Table 3) and tested their antimicrobial activities against *E. coli*, *S. aureus* and *C. albicans* using a radial diffusion assay (Figure 3). We found antimicrobial activity in 13 synthetic peptides (Ph 1, 2, 3, 4, 8, 10, 12, 14, 16, 20, 22, 24, 26), which increased in a dose-dependent manner. Remarkably, the peptides Ph 1, 2, 22, 26 showed stronger antimicrobial activity than melittin and Ph 3, 4 showed similar antimicrobial activity with melittin in *E. coli*. The antimicrobial activities of Ph 22 showed similar antimicrobial activity with melittin in *S. aureus*. Correspondingly, Ph 2, 3, 14, 22 showed stronger antimicrobial activity than melittin and Ph 1, 16, 20 showed similar antimicrobial activity with melittin against *C. albicans* (Table 4). The results showed that Ph 22 had a broad range of activities toward Gram-negative bacteria, Gram-positive bacteria, and yeast. 

**Figure 4:**
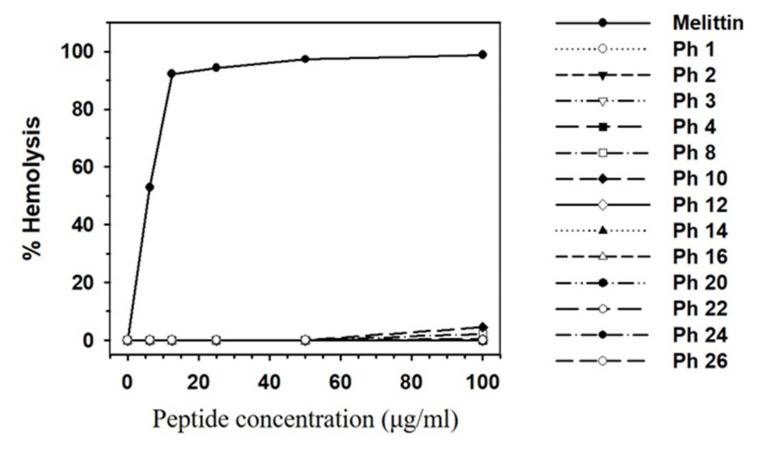
Cytotoxic effects of 13 selected peptides. Hemolytic activity of the peptides. Peptide concentration (*x*-axis) is plotted against the percentage of hemolysis (*y*-axis) of mouse red blood cells after incubation for 30 min. Melittin was used as the positive control. The percent hemolysis was calculated with the following equation: hemolysis (%) = (A540 of samp—A540 of peptide-free control)/(A540 of 100% control—A540 of peptide-free control) × 100. Each symbol represents the mean value estimated from triplicate experiments.

The hemolytic effects of the 13 selected synthetic peptides showing antimicrobial activity in the radial diffusion assay were shown in Figure 4. Melittin lysed 92% of mouse red blood cells at a concentration of 12.5 μg/mL, whereas no or little hemolytic activity was observed for the 13 synthetic peptides at this concentration and up to 100 μg/mL. Melittin has strong and broad antimicrobial spectrum, but the peptide lacks selectivity in normal cells. The purpose of this experimental study is to find novel peptides, which have potent antimicrobial activities with little or no cytotoxicity. Thus, these data indicate that the selected peptides are useful for the development of novel antimicrobial agents.

## 4. Conclusions

Insects are rich sources of AMPs with broad range therapeutic potential against pathogenic microbes. In this study we chose *P. hilaris*, the yellow spotted long horned beetle and immunized it with a bacterial and fungal strain. A potential AMP candidate was then obtained from the immune challenged transcriptome. The observed antimicrobial activity of these AMPs with further validation could serve as new curative for multidrug resistant microbes.

## Figures and Tables

**Table 1:** Summary of sequencing and annotation.

Name	Raw Reads	Size (GB)	Clean Reads	De Novo Alignment (%)
PH_N1 ^1^	22,668,170	6	22,247,657	81.36
PH_N2 ^1^	23,223,996	6.9	22,706,277	81.48
PH_N3 ^1^	22,065,414	7.3	21,617,382	81.16
PH_I1 ^2^	19,915,844	6.8	19,547,003	86.32
PH_I2 ^2^	22,838,132	7	22,388,341	85.36
PH_I3 ^2^	24,414,115	6.6	23,931,670	83.60
**De novo assembly** Unigenes Base pairs Average length of unigenes GC percentage **Annotation** **Descriptions** BLAST- Swissprot No blast Gene Ontology	35,715 73,299,234 2052 37.53 **No of hits** 33,932 1783 26,492	**Percentage** 95.0 5.0 74.0		

^1^ Non-immunized, ^2^ Immunized, GB—Gigabyte, GC—Guanine-cytosine.

**Table 2:** Antimicrobial peptide properties prediction and filtration.

Propensity	Methods	Cutoff	No. of Peptide Sequences
	Raw Sequence	Total peptides (2 to 50 amino acids)	104,615
Molecular	Pepstats Pepstats AMPA	Charge > 0 (+) 8 ≥ pI ≤ 12 Stretch no ≥1	70,364 51,494 83,783
Aggregation (Invivo)	Tango Tango Tango	AGG (≤500) Helix (≥0 helix ≤25) Beta (≥25 beta ≤100)	85,941 98,871 48,068
Aggregation (Invitro)	Aggrescan	Na4VSS (≥−40 Na4vSS ≤60)	80,821
Similarity	BlastP (E value: 1E-05)	Known AMPs (ADAM, >80) Known AMPs (APD, >80) Known AMPs (CAMP, >80) Novel AMPs (Similarity <80)	0 0 0 14,405
AMP	CAMP CAMP CAMP CAMP ADAM	SVM (>0.5, AMP) RF (>0.5, AMP) ANN (AMP) DA (>0.5, AMP) SVM (>0.5, AMP)	4653 5019 7363 5655 10,590
Final			2290

ADAM—A Database of Anti-Microbial peptides, APD—Antimicrobial Peptide Database, ANN—Artificial Neural Network.

**Table 3:** Primary sequences of *P. hilaris* peptides.

Peptide	Protein Length	CAMP-SVM Score	CAMP-RF Score	CAMP-DA Score	ADAM-SVM Class	Peptide Sequence
Ph 1	16	0.793	0.8945	0.732	1.63	RAIKWPGNGLLFLKY *
Ph 2	15	0.701	0.976	0.959	1.84	KLPIVNVKLVNRIK *
Ph 3	14	0.775	0.688	0.784	1.2	KRGYQVPRIAFII *
Ph 4	16	0.674	0.6385	0.965	1.2	KLQVVPAIHLVWLQK *
Ph 8	14	0.832	0.7725	0.746	1.63	RCLKTCFLSFIRY *
Ph 10	13	0.555	0.816	0.855	0.45	RISCVAMRLILK *
Ph 12	13	0.551	0.6325	0.902	2.53	RLLLLCYACGKS *
Ph 14	10	1	0.627	0.989	3.49	RRRCRCCRY *
Ph 16	10	0.999	0.6585	0.833	2.15	RKSWRHWKC *
Ph 20	13	0.667	0.6365	0.751	1.2	KMFTKCIRYRKM *
Ph 22	12	0.93	0.621	0.554	1.06	KRIFYLYIRGQ *
Ph24	14	0.706	0.7095	0.595	1.56	KLSNAVFKSCRKI *
Ph26	14	0.898	0.534	0.788	0.72	KIGTFIKKLSYTS *

* Signify C—terminal amidation, CAMP—Collection of Anti-Microbial Peptides, SVM—Support vector machine, RF—Random Forest, DA—Discriminant Analysis.

**Table 4:** Antimicrobial activities of *P. hilaris* peptides.

Peptides (200 μg/mL)	*E. coli*	*S. aureus*	*C. albicans*
Melittin	75	100	65
Ph 1	85	86	65
Ph 2	80	90	80
Ph 3	72	85	80
Ph 4	75	80	30
Ph 8	71	83	45
Ph 10	67	72	48
Ph 12	70	75	40
Ph 14	65	70	105
Ph 16	50	87	65
Ph 20	65	85	68
Ph 22	85	95	92
Ph 24	49	65	47
Ph 26	85	75	25

Diameters of clearing zone have been expressed in units (1 mm = 10 Units).

## References

[1] Li J., Koh J.J., Liu S., Lakshminarayanan R., Verma C.S., Beuerman R.W. (2017). Membrane Active Antimicrobial Peptides: Translating Mechanistic Insights to Design. Front. Neurosci..

[2] Fry D.E. (2018). Antimicrobial Peptides. Surg. Infect..

[3] Haney E.F., Mansour S.C., Hancock R.E. (2017). Antimicrobial Peptides: An Introduction. Methods Mol. Biol..

[4] Brady D., Grapputo A., Romoli O., Sandrelli F. (2019). Insect Cecropins, Antimicrobial Peptides with Potential Therapeutic Applications. Int. J. Mol. Sci..

[5] Yi H.Y., Chowdhury M., Huang Y.D., Yu X.Q. (2014). Insect antimicrobial peptides and their applications. Appl. Microbiol. Biotechnol..

[6] Tonk M., Vilcinskas A. (2017). The Medical Potential of Antimicrobial Peptides from Insects. Curr. Top. Med. Chem..

[7] Wu Q., Patocka J., Kuca K. (2018). Insect Antimicrobial Peptides, a Mini Review. Toxins.

[8] Kolmar H. (2009). Biological diversity and therapeutic potential of natural and engineered cystine knot miniproteins. Curr. Opin. Pharmacol..

[9] Gracy J., Chiche L. (2011). Structure and modeling of knottins, a promising molecular scaffold for drug discovery. Curr. Pharm. Des..

[10] Tam J.P., Wang S., Wong K.H., Tan W.L. (2015). Antimicrobial Peptides from Plants. Pharmaceuticals.

[11] Hwang J.S., Lee J., Hwang B., Nam S.H., Yun E.Y., Kim S.R., Lee D.G. (2010). Isolation and characterization of Psacotheasin, a novel Knottin-type antimicrobial peptide, from *Psacothea hilaris*. J. Microbiol. Biotechnol..

[12] Hwang B., Hwang J.S., Lee J., Lee D.G. (2010). Antifungal properties and mode of action of psacotheasin, a novel knottin-type peptide derived from *Psacothea hilaris*. Biochem. Biophys. Res. Commun..

[13] Hwang B., Hwang J.S., Lee J., Lee D.G. (2011). The antimicrobial peptide, psacotheasin induces reactive oxygen species and triggers apoptosis in *Candida albicans*. Biochem. Biophys. Res. Commun..

[14] Yoo W.G., Lee J.H., Shin Y., Shim J.Y., Jung M., Kang B.C., Oh J., Seong J., Lee H.K., Kong H.S. (2014). Antimicrobial peptides in the centipede Scolopendra subspinipes mutilans. Funct. Integr. Genomics.

[15] Kim I.W., Lee J.H., Subramaniyam S., Yun E.Y., Kim I., Park J., Hwang J.S. (2016). De Novo Transcriptome Analysis and Detection of Antimicrobial Peptides of the American Cockroach *Periplaneta americana* (Linnaeus). PLoS ONE.

[16] Kim I.W., Markkandan K., Lee J.H., Subramaniyam S., Yoo S., Park J., Hwang J.S. (2016). Transcriptome Profiling and In Silico Analysis of the Antimicrobial Peptides of the Grasshopper *Oxya chinensis sinuosa*. J. Microbiol. Biotechnol..

[17] Bolger A.M., Lohse M., Usadel B. (2014). Trimmomatic: A flexible trimmer for Illumina sequence data. Bioinformatics.

[18] Haas B.J., Papanicolaou A., Yassour M., Grabherr M., Blood P.D., Bowden J., Couger M.B., Eccles D., Li B., Lieber M. (2013). De novo transcript sequence reconstruction from RNA-seq using the Trinity platform for reference generation and analysis. Nat. Protoc..

[19] Li W., Godzik A. (2006). Cd-hit: A fast program for clustering and comparing large sets of protein or nucleotide sequences. Bioinformatics.

[20] Young M.D., Wakefield M.J., Smyth G.K., Oshlack A. (2010). Gene ontology analysis for RNA-seq: Accounting for selection bias. Genome Biol..

[21] Langmead B., Trapnell C., Pop M., Salzberg S.L. (2009). Ultrafast and memory-efficient alignment of short DNA sequences to the human genome. Genome Biol..

[22] Robinson M.D., Oshlack A. (2010). A scaling normalization method for differential expression analysis of RNA-seq data. Genome Biol..

[23] Li B., Dewey C.N. (2011). RSEM: Accurate transcript quantification from RNA-Seq data with or without a reference genome. BMC Bioinformatics.

[24] Robinson M.D., McCarthy D.J., Smyth G.K. (2010). edgeR: A Bioconductor package for differential expression analysis of digital gene expression data. Bioinformatics.

[25] Lee H.T., Lee C.C., Yang J.R., Lai J.Z., Chang K.Y. (2015). A large-scale structural classification of antimicrobial peptides. Biomed Res. Int..

[26] Wang G., Li X., Wang Z. (2016). APD3: The antimicrobial peptide database as a tool for research and education. Nucleic. Acids Res..

[27] Waghu F.H., Gopi L., Barai R.S., Ramteke P., Nizami B., Idicula-Thomas S. (2014). CAMP: Collection of sequences and structures of antimicrobial peptides. Nucleic. Acids Res..

[28] Steinberg D.A., Lehrer R.I. (1997). Designer assays for antimicrobial peptides. Disputing the “one-size-fits-all” theory. Methods Mol. Biol..

